# Autotoxicity and Allelopathy of 3,4-Dihydroxyacetophenone Isolated from *Picea schrenkiana* Needles

**DOI:** 10.3390/molecules16108874

**Published:** 2011-10-24

**Authors:** Xiao Ruan, Zhao-Hui Li, Qiang Wang, Cun-De Pan, De-An Jiang, G. Geoff Wang

**Affiliations:** 1 College of Life Science, Zhejiang University, Hangzhou, 310058, China; 2 Ningbo Institute of Technology, Zhejiang University, Ningbo, 315100, China; 3 College of Forestry, Xinjiang Agricultural University, Urumqi, 830052, China; 4 Department of Forestry and Natural Resources, Clemson University, 261 Lehotsky Hall, Clemson, SC 29634, USA

**Keywords:** *Picea schrenkiana* Fisch. et Mey., allelochemicals, 3,4-dihydroxy acetophenone (DHAP), germination rate, germination vigor, seedling growth

## Abstract

Bioassay-guided fractionation of the diethyl ether fraction of a water extract of* Picea schrenkiana* needles led to the isolation of the phenolic compound 3,4-dihydroxy- acetophenone (DHAP). The allelopathic effects of DHAP were evaluated under laboratory conditions on* P. schrenkiana*, rice (*Oryza sativa* L.), wheat (*Triticum aestivum* L.), radish (*Raphanus sativus* L.), lettuce (*Latuca sativa* L.), cucumber (*Cucumis sativus* L.) and mung bean (*Phaseolus radiatus* L.). DHAP significantly inhibited seed germination and seedling growth of *P. schrenkiana* at concentrations of 2.5 mM and 0.5 mM (*p* < 0.05). Soil analysis revealed that *P. schrenkiana* forest soils contained exceptionally high DHAP concentrations (mean = 0.51 ± 0.03 mg/g dry soil), sufficient to inhibit natural *P. schrenkiana* recruitment. DHAP also exhibited strong allelopathic potential. It significantly inhibited wheat and lettuce seed germination at concentrations of 1 mM and 0.5 mM (*p* < 0.05). The active compound also completely inhibited root growth of the six test species at high concentrations. Our results suggest a dual role of DHAP, both as an allelochemical and as an autotoxicant. The potential for a single plant needle-leached compound to influence both inter- and intra-specific interactions emphasized the complex effects that plant secondary metabolites might have on plant population and community structure.

## 1. Introduction

Plants synthesize an array of chemicals that are involved a variety of plant-plant, plant-microbe, and plant-herbivore interactions [1,2]. The allelochemicals responsible for plant-plant allelopathy are delivered into the environment mainly through decomposition, leaching, volatilization or root exudation [3,4]. Allelopathy is usually interspecific [5,6], but also may occur within the same species, which is called autotoxicity [7]. Autotoxicity, defined as the deleterious allelopathic effect among the individuals of the same species, has been documented in a number of coniferous species, and it is believed to be involved in natural and managed ecosystems. The problem of autotoxicity is common in woodlands, and is one of the major reasons for growth reduction under the continuous monculture practice. In forest ecosystems, many examples of autotoxicity exist in coniferous trees, including *Abies balsamea* [8], *Cunninghamia lanceolata* [9], *Picea abies* [10], *Picea mariana* [11], *Pinus halepensis* [12,13,14], *Pinus densiflora* [15] and *Pinus laricio* [16]. Autotoxicity plays an important role in natural and managed coniferous forest ecosystems, often causing problems in natural or artifical regeneration [17,18,19].

Schrenk spruce (*Picea schrenkiana* Fisch. et Mey.), the most important zonal vegetation of Tianshan Mountains in China, is an endemic species in Middle Asia and the mountains of Asia. In China, it is mainly distributed on the northern and southern slopes of Tianshan Mountains, and the northern slope of the western part of Kunlun Mountains, accounting for half of the woodland area in Xinjiang province, with an area of 528,400 hm^2^ [20]. As a major tree species in the forest ecosystems of Xinjiang, *P. schrenkiana* plays an important role in water conservation. However, natural regeneration has been problematic, which has been widely documented. It has been hypothesized that secondary metabolites released by litter and root secretion accumulates in the rhizosphere due to fire suppression [21], and these accumulated chemicals are autotoxic to the regeneration of *P. schrenkiana*.

Previous results showed that the original water extract of *P. schrenkiana* needles and the diethyl ether, ethyl acetate and *n*-butanol soluble fractions of the original water extract all exhibited strong autotoxic effects on seed germination and seedling growth [22,23]. Investigation of the chemical composition of *P. schrenkiana* needles reveals a great number of secondary metabolites that may serve as allelochemicals. Among them, phenolic acids, long-chain fatty acid, tannin, indole and flavonoid were best correlated to the observed autotoxic effects. These compounds were frequently identified from pine needles, bark and soils under the pine trees as putative allelopathic substances [24,25]. Seed germination and growth inhibition by phenolic acids and other allelochemicals had been widely observed [26,27,28].

In this study, we further investigated the allelopathic potential of *P. schrenkiana* needles. We first isolated and identified the active compound from the extract of *P. schrenkiana* needles. We then tested the allelopathic and autotoxic effect of this active compound on seed germination and seedling growth by conducting a laboratory bioassay on *P. schrenkiana* and six common crop species.

## 2. Results

### 2.1. Identification and Quantitation Analysis of DHAP

**Figure 1:**
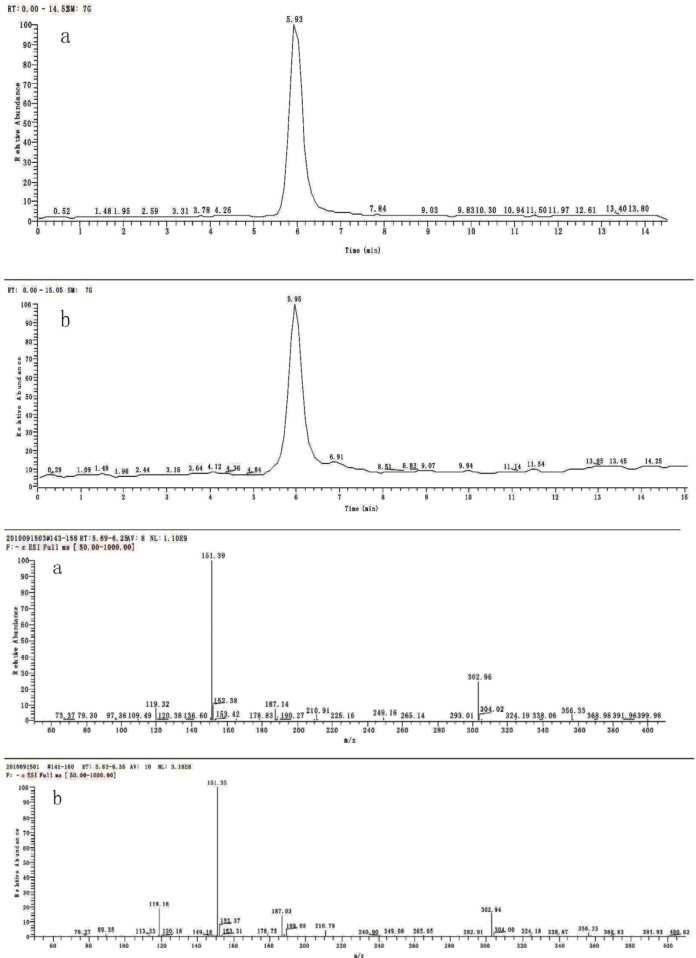
The result of LC-MSD analysis (a: crystal sample; b: DHAP authentic standard).

According to the LC-MS data (Figure 1), the compound was identified as a phenolic compound, 3,4-dihydroxyacetophenone. This compound was first reported by Beijing Pharmaceutical Research Institute, 1977, as a herbal medicine ingredient [29]. The compound is indefinitely stable at room temperature, the average half life and biological degradation in soil of the compound have not been reported.

The linear calibration curve for HPLC analysis of DHAP in* P. schrenkiana* needles fits the equation Y = 5275.2X + 166.73 (r^2^ = 0.99787; n = 15—The number of points in the calibration curve, representing five different concentrations and determined three times each; Y = peak height ratio; X = concentration). The mean (±SD) concentration of DHAP in *P. schrenkiana* needles and ten field soil samples were 4.93 ± 0.41 mg/g and 0.51 ± 0.03 mg/g dry weight, which were determined by HPLC analysis.

**Figure 2:**
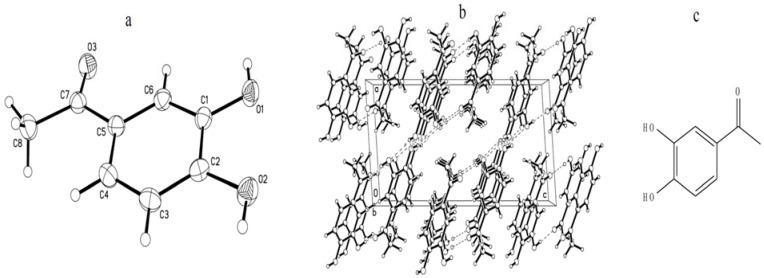
Chemical structure information of crystal sample (a: Crystal structure; b: Stacked crystal structure; c: molecule structure).

Finially, the structure was confiomed by X-ray crystallography (Figure 2). CCDC 791512 contains the supplementary crystallographic data for this paper. These data can be obtained free of charge via www.ccdc.cam.ac.uk/conts/retrieving.html (or from the CCDC, 12 Union Road, Cambridge CB2 1EZ, UK; Fax: +44-1223-336033; e-mail: deposit@ccdc.cam.ac.uk).

### 2.2. Effects of DHAP on Seed Germination

DHAP affected *P. schrenkiana*, rice, wheat and lettuce seed germination when measured as either germination rate or germination vigor (Table 1). Seed vigor is defined as the sum total of those properties of the seed that determine the level of activity and performance of the seed during germination and seedling emergence. The concept of seed vigor is of vital importance to the seed industry because two seed lots with same germination percentage, but differing vigor, could show significant variation in stand and yield when planted under various stress conditions [46]. DHAP significantly inhibited *P. schrenkiana* seed germination rate at concentrations of 2.5 mM (*p* < 0.05). Germination vigor was inhibited at concentrations of 1.0 mM (*p* < 0.05). The seed germination vigor was zero at 10 mM (*p* < 0.05), indicating there was no seed germinated until 7 days after treatment. The autotoxic threshold concentration of DHAP on *P. schrenkiana* seed germination is 2.5 mM, at which the germination rate and germination vigor were only 83.56% and 83.87% of the control. The inhibitory effect was concentration dependent and the growth declined with increasing concentration, indicating dose-response behavior.

DHAP significantly inhibited wheat seed germination rate and germination vigor at concentrations of 1 mM (*p* < 0.05) (Table 1). The inhibitory threshold concentration of DHAP on wheat seed germination is 1 mM, at which the germination rate and germination vigor were only 95.67% and 92.3% of the control. DHAP also significantly inhibited lettuce seed germination rate at concentrations of 0.5 mM (*p* < 0.05) (Table 1). Germination vigor was inhibited at concentrations of 0.5 mM (*p* < 0.05). The inhibitory threshold concentration of DHAP on lettuce seed germination is 0.5 mM, at which the germination rate and germination vigor were only 84.68% and 78.37% of the control.

For all the six treatment solutions, DHAP had no inhibitory effects on rice seed germination (Table 1). However, the compound at 1 and 10 mM concentration significantly promoted rice seed germination, and the germination vigor were up to 147.94% and 166.69% of control. The six treatment solutions of DHAP had no negative or positive effects on radish, cucumber and mung bean seed germination (Table 1).

**Table 1:** Effects of DHAP on seed germination.

Plant	Concentration mM	Germination Rate (% of control)	Germination vigor (% of control)
*P. schrenkiana*	0	100 a	100 a
	0.1	98.13 ± 4.18 a	91.39 ± 6.03 a
	0.5	94.37 ± 6.25 a	97.84 ± 1.53 a
	1	96.24 ± 4.16 a	86.03 ± 0.58 ab
	2.5	83.56 ± 2.52 b	83.87 ± 1.73 b
	5	85.45 ± 7.02 b	23.65 ± 0.58 c
	10	74.18 ± 5.13 c	0 ± 0 d
Wheat	0	100 a	100 a
	0.1	99.33 ± 0.58 a	98.66 ± 0.58 a
	0.5	97.67 ± 0.58 ab	96.65 ± 1.53 ab
	1	95.67 ± 1.53 b	92.30 ± 1.00 b
	2.5	92.33 ± 4.93 cd	90.30 ± 6.08 bc
	5	94.00 ± 1.00 c	91.30 ± 0 b
	10	89.33 ± 2.08 d	81.60 ± 5.51 c
Lettuce	0	100 a	100 a
	0.1	98.08 ± 3.22 a	88.10 ± 8.74 a
	0.5	84.68 ± 0.58 b	78.37 ± 4.51 bc
	1	81.23 ± 1.07 b	75.13 ± 8.51 c
	2.5	81.23 ± 3.22 b	71.35 ± 5.29 cd
	5	78.93 ± 6.11 bc	62.70 ± 4.62 de
	10	63.60 ± 7.02 c	42.16 ± 6.08 e
Rice	0	100 a	100 a
	0.1	97.56 ± 1.86 a	106.25 ± 2.65 c
	0.5	97.06 ± 3.00 a	112.50 ± 3.61 c
	1	96.53 ± 4.36 a	147.94 ± 0.55 b
	2.5	97.76 ± 1.45 a	131.25 ± 1.61 bc
	5	96.76 ± 3.46 a	104.19 ± 1.53 c
	10	97.59 ± 3.00 a	166.69 ± 2.08 a
Radish	0	100 a	100 a
	0.1	98.97 ± 1.53 a	96.83 ± 5.77 a
	0.5	97.27 ± 0.58 a	94.36 ± 2.08 a
	1	97.60 ± 4.58 a	97.18 ± 5.19 a
	2.5	96.24 ± 2.31 a	94.36 ± 3.22 a
	5	100.00 ± 1.53 a	97.53 ± 1.53 a
	10	97.95 ± 1.53 a	97.53 ± 0.58 a
Cucumber	0	100 a	100 a
	0.1	96.10 ± 2.08 a	96.83 ± 5.77 a
	0.5	96.46 ± 4.51 a	94.36 ± 2.08 a
	1	98.59 ± 4.04 a	97.18 ± 5.19 a
	2.5	96.46 ± 2.52 a	94.36 ± 3.22 a
	5	97.52 ± 3.51 a	97.53 ± 1.53 a
	10	96.10 ± 4.51 a	97.53 ± 0.58 a

Means within a column followed by the same letter are not different at P = 0.05 level according to Fisher’s test; each point is the mean of three replicates ± s.d.

### 2.3. Effects of DHAP on Seedling Growth

**Figure 3:**
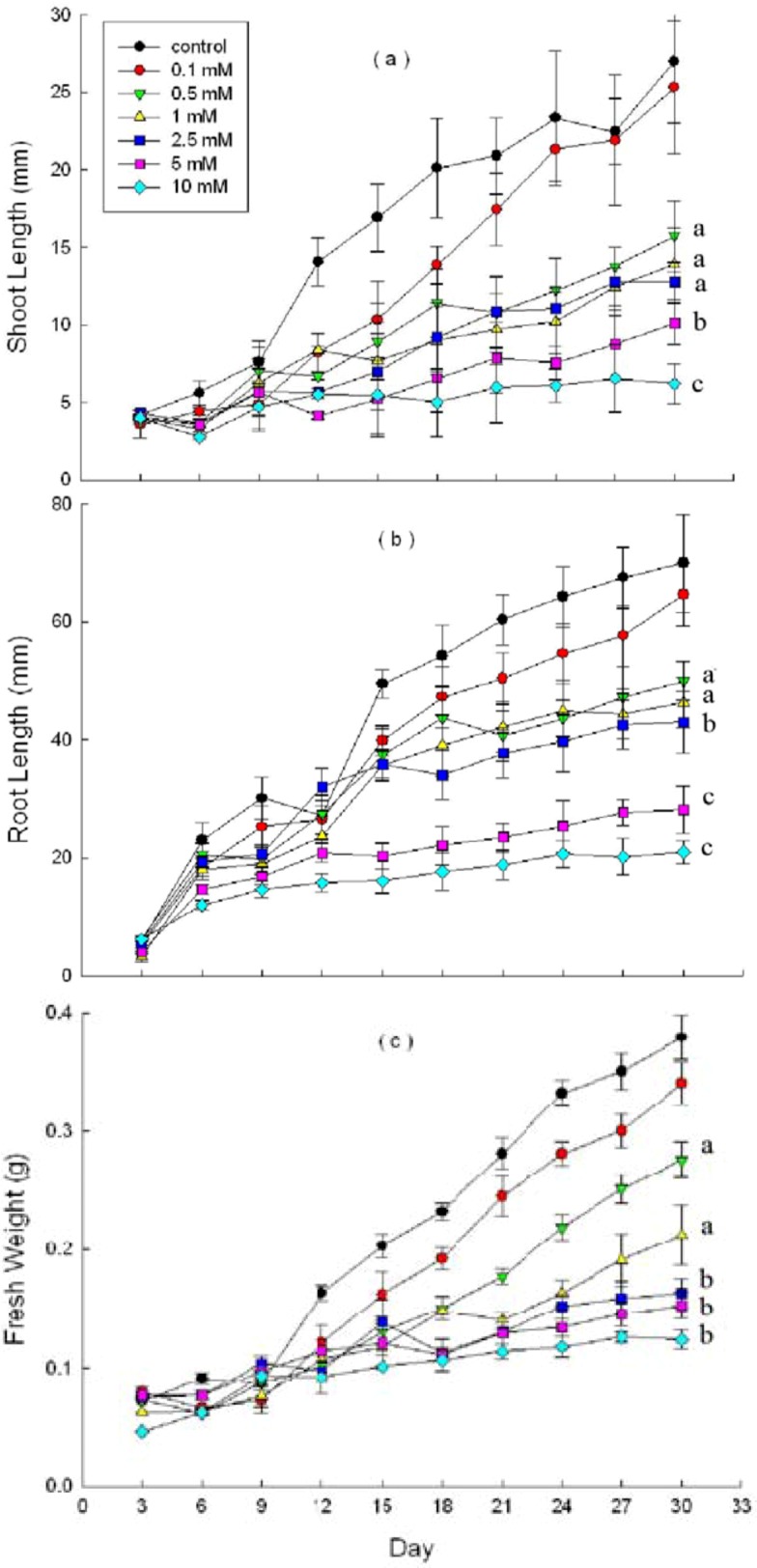
Effects of DHAP on shoot length (**a**), root length (**b**) and fresh weight (**c**) of *P. schrenkiana*; Line graphs within the same plot followed by the same letter are not different at P = 0.05 level according to Fisher’s test; Each point is the mean of three replicates ± s.d.

DHAP severely affected shoot and root growth potential of *P. schrenkiana* seedlings. The average shoot and root length of seedlings were shorter after DHAP treatment (except at 0.1 mM). This inhibitory effect was concentration dependent (Figure 3). There was a similar trend of changes in the fresh weight parameter. Root growth decreased significantly (*p* < 0.05 at 2.5 mM) in response to DHAP treatments.

Compared with root length, the decrease in shoot length was less, with significant effects observed at 5 mM (*p* < 0.05) DHAP. The decrease in fresh weight was the least, with significant effects found at 2.5 mM (*p* < 0.05) DHAP. The autotoxic concentration threshold of DHAP on *P. schrenkiana* seedling growth is 2.5 mM. In addition, high DHAP concentrations (≥5 mM) resulted in dark brown *P. schrenkiana* roots among some seedlings.

**Figure 4:**
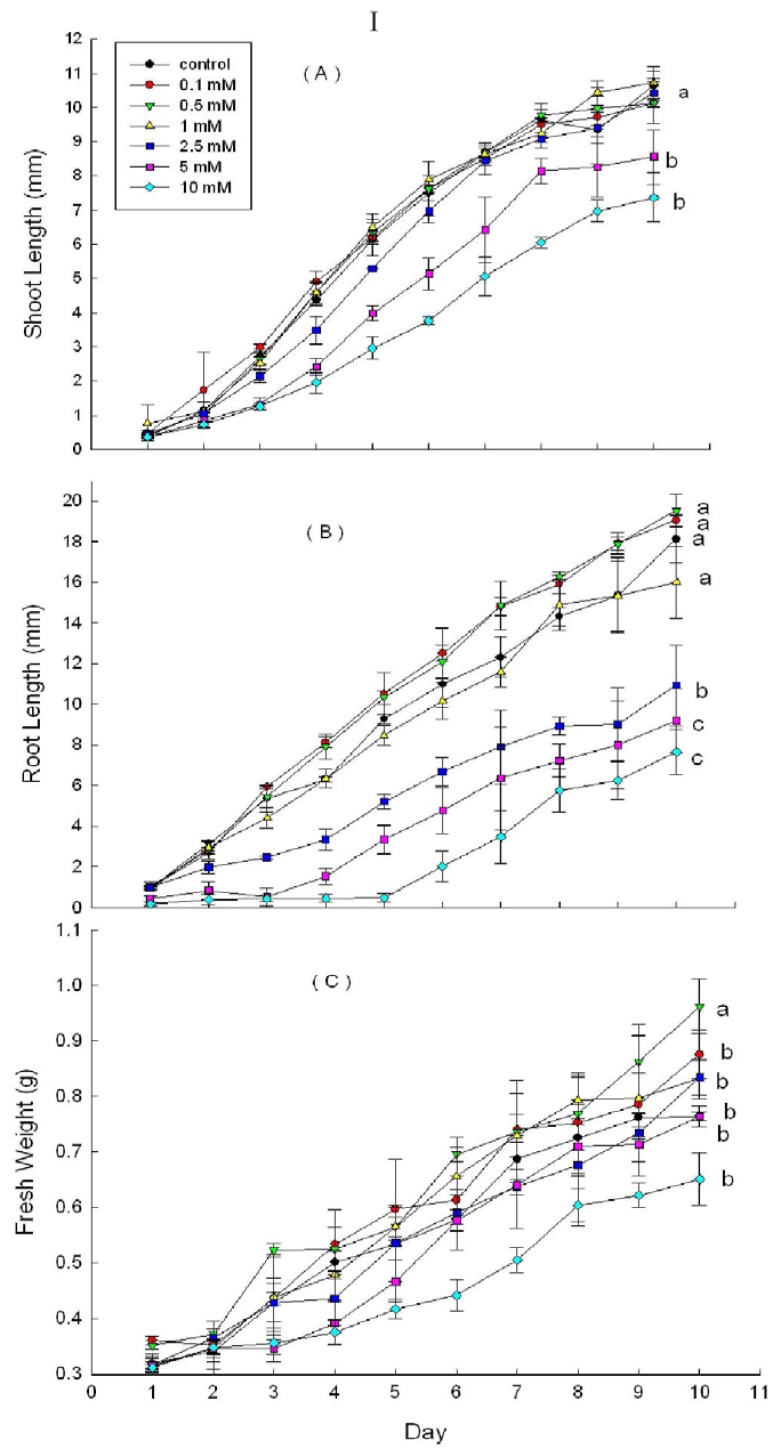

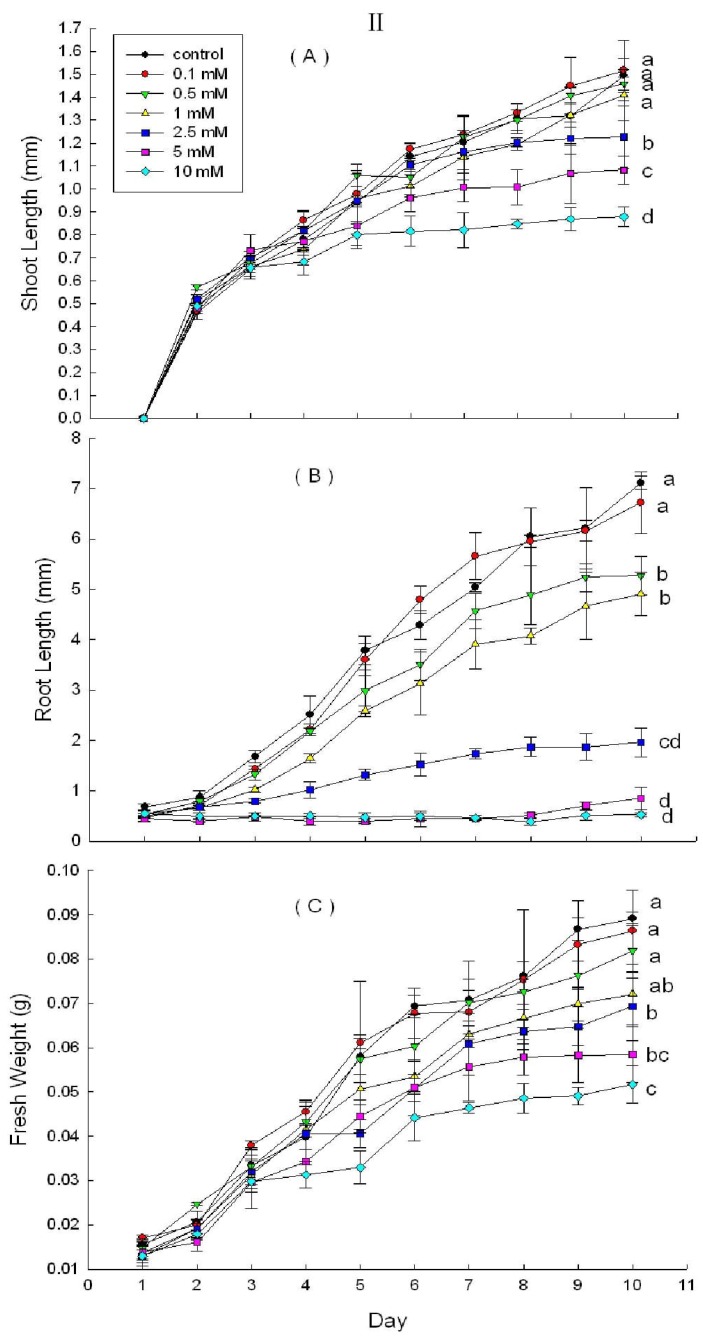

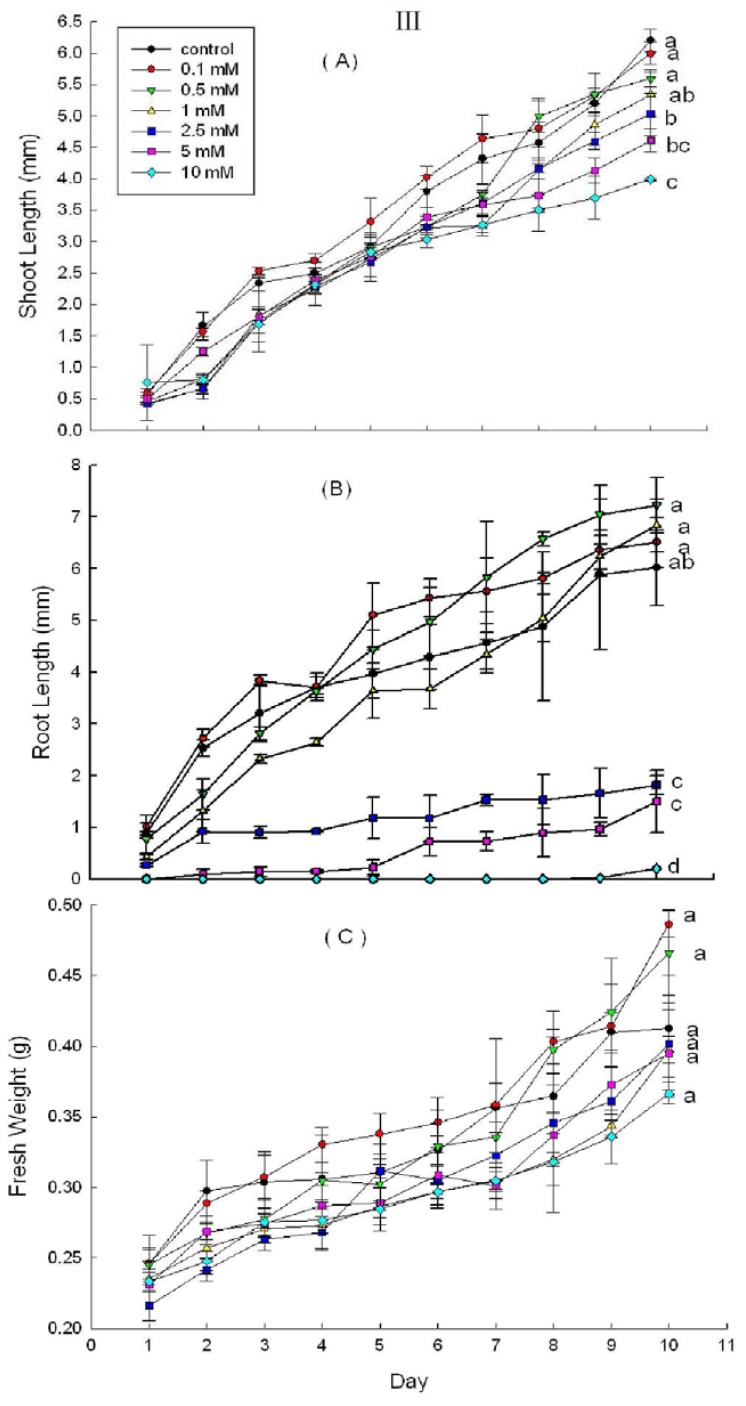
Effects of DHAP on shoot length (**a**), root length (**b**) and fresh weight (**c**) of wheat (I), lettuce (II) and rice (III); Line graphs within the same plot followed by the same letter are not different at P = 0.05 level according to Fisher’s test; Each point is the mean of three replicates ± s.d.

DHAP treatment solutions significantly inhibited wheat root growth (*p* < 0.05 at 2.5 mM) and shoot growth at 5 mM. DHAP at 0.5 mM had significantly promoted wheat growth, resulting in higher fresh weight (Figure 4, I).

**Figure 5:**
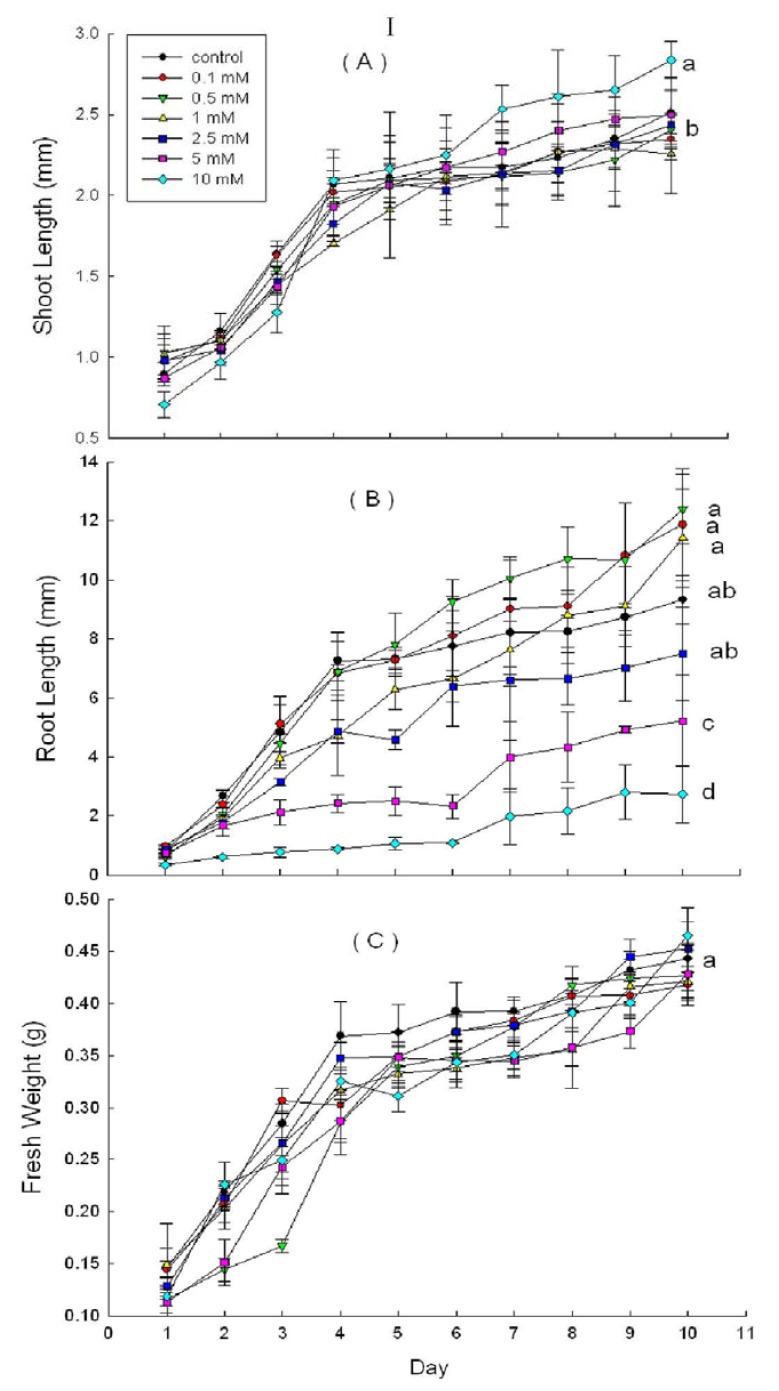

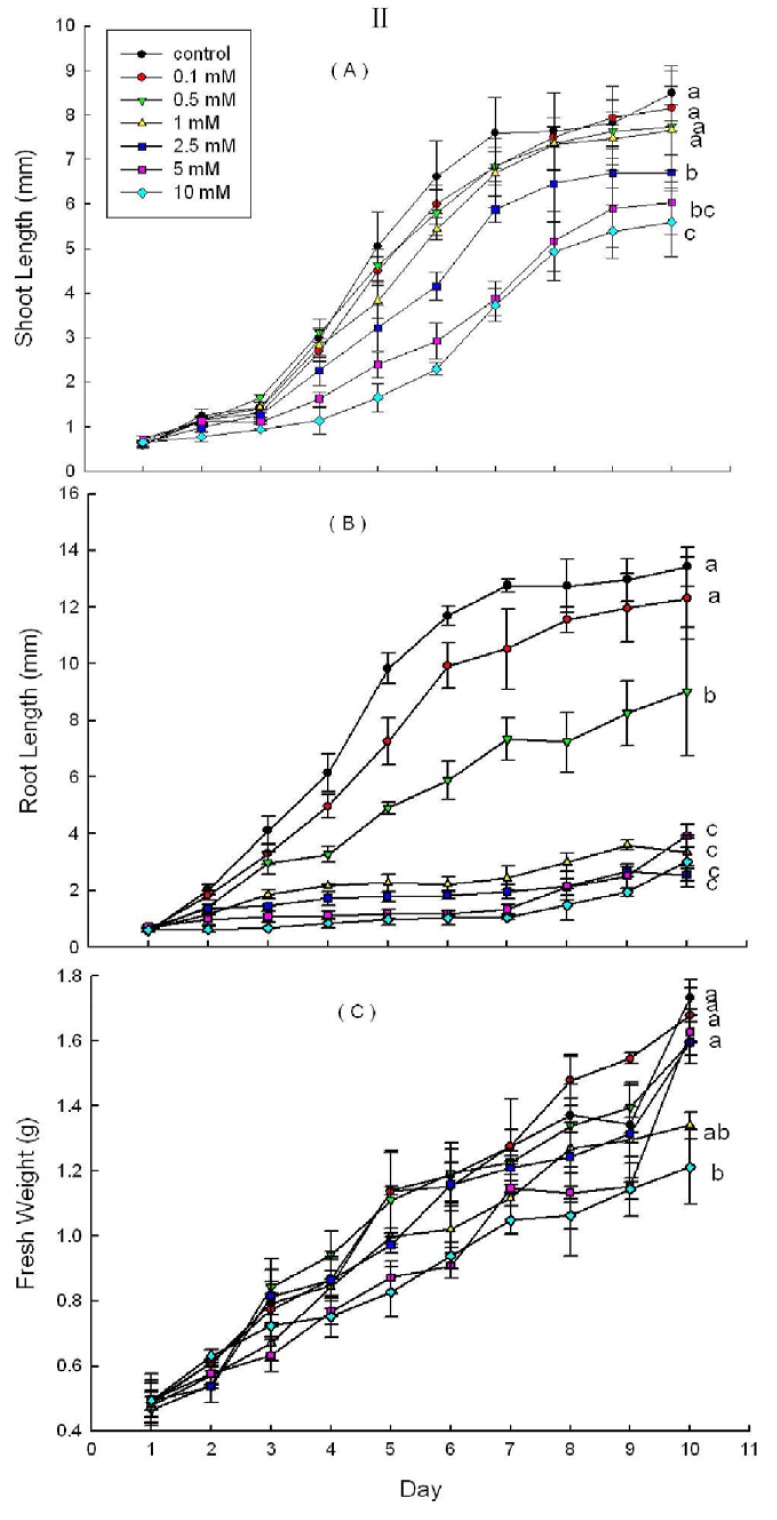

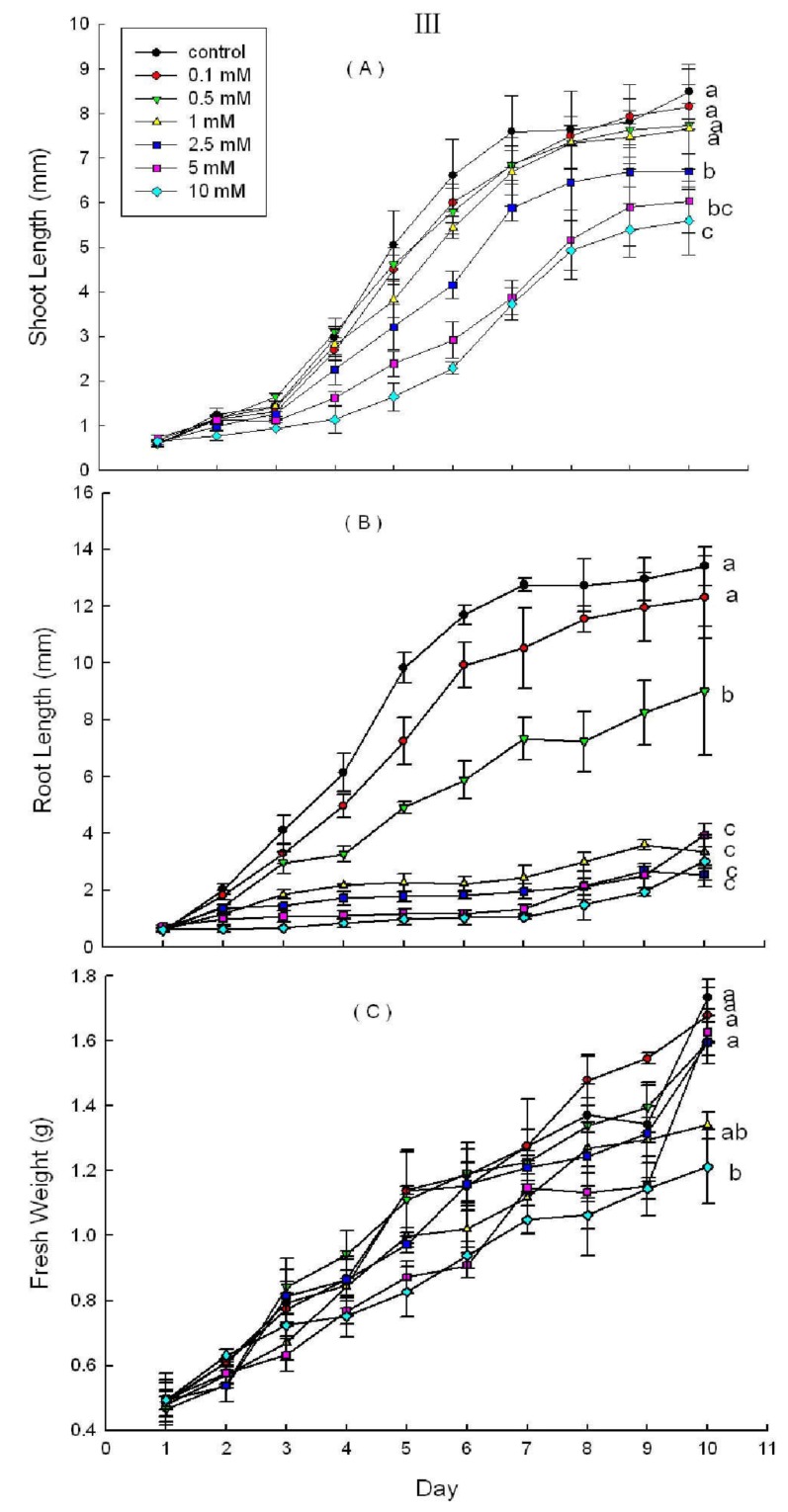
Effects of DHAP on shoot length (**a**), root length (**b**) and fresh weight (**c**) of radish (I), cucumber (II) and mung bean (III); Line graphs within the same plot followed by the same letter are not different at P = 0.05 level according to Fisher’s test; Each point is the mean of three replicates ± s.d.

DHAP at 10 mM concentration could significantly promoted radish shoot growth (Figure 5, I). DHAP treatment solutions significantly inhibited cucumber root growth at 0.5 mM (Figure 5, II). DHAP treatment solutions significantly inhibited mung bean root growth at 0.5 mM), shoot growth at 2.5 mM, and fresh weight at 10 mM (Figure 5, III).

## 3. Discussion

DHAP, an active ingredient of traditional Chinese medicine, was first isolated from *Ilex pubescens* Hook. et Arn. var. glaber Cheng [29]. In addition to antimelanogenic activity, the compound is also useful in treating pathologic pregnancies with chronic defective utero-placental circulation such as PIH and IUGR [30,31]. However, our study is the first to report that DHAP inhibits plant growth. Our results demonstrated that DHAP acted both as an autotoxicant and allelochemical because of its intraspecific and interspecific effects. Accumulation of DHAP in *P. schrenkiana* forest soils may have a negative impact on *P. schrenkiana* natural regeneration.

Higher plants release a diversity of allelochemicals into the environment, including phenolics, alkaloids, long chain fatty acids, terpenoids and flavonoids [3,32,33,34]. Phenolics mixtures: vanillic, benzoic, protocatechuic, cinnamic, syringic and ferulic acids extracted from litters and rhizosphere soil of *Pinus laricio* were most inhibitory to its seed germination [16]. *p*-Hydroxybenzoic, gallic, coumaric, ferulic, vanillic and protocatechuic acids were the allelochemicals responsible for autotoxicity in replanted Chinese fir [*Cunninghamia lanceolata* (Lamb.) Hook] trees [35,36,37]. Autotoxic components analysis of *Pinus halepensis* also showed phenolic compounds (4-hydroxybenzoic, vanillic, syringic, gallic, *p*-coumaric, benzoic, cinnamic, ferulic acid and caffeic acids) in aqueous extracts [12,38]. In this study, we isolated a phenolic autotoxic substance, DHAP, from the diethyl ether fraction of the water extract of *P. schrenkiana* needles as the major active compound responsible for the observed autotoxic and allelopathic effects.

Seed germination and seedling growth studies using phytochemical extracts are most widely used to determine the allelopathic potential in vegetation [39,40]. Crop seeds are commonly selected for use in phytotoxic bioassays, because they satisfy a number of selection criteria: They are readily available, affordable, repeatable and reliable; and they germinate quickly, completely, and uniformly. In this study, we selected six crops as test species. DHAP showed inhibitory effects on both dicotyledonous plant species (lettuce, radish, cucumber and mung bean) and monocotyledonous plant species (wheat and rice) (Table 1, Figure 4 and Figure 5). Our results indicated that DHAP could inhibit seed germination of wheat and lettuce, and seedling root growth of all the six crop species.

It was known that some compounds act as plant growth regulators, exhibiting hormesis, or concentration-dependent stimulatory or inhibitory effects on seedling growth [15,41,42]. Weir *et al.* [19] discovered that (−)-catechin isolated from *Centaurea maculosa* stimulated roots growth in *Gaillardia aristata* and *Lobelia erinus* at 10 µg/mL, but had a significant inhibitory effect at 400 µg/mL. Needle-leached DHAP had a similar effect on some of the plants that we tested, showing increased growth of radish shoot and rice root at lower concentrations of DHAP. In addition, root growth inhibition of the six crop species treated with ≥5 mM of DHAP indicate that this phytochemical could act as a plant growth regulator. The results for root and shoot growth inhibition indicated that the inhibitory effect of DHAP was greater on root growth than shoot growth of all the bioassay seedlings. A similar conclusion was reached for other phenolic compounds [43,44,45]. This can be attributed to the fact that roots are the first to sense and respond to allelochemicals from the environment.

The field soil samples from a mature *P. schrenkiana* forest contained 0.51 ± 0.03 mg DHAP/g dry soil. Our laboratory autotoxicity bioassays results showed that DHAP at 2.5 mM significantly inhibited *P. schrenkiana* seed germination and seedling growth (Table 1 and Figure 3). If DHAP in 1 g field soil were dissolved into 1 mL soil water, the concentration of DHAP would be 0.51 mg/mL. Considering the threshold of DHAP was 2.5 mM for seed germination and growth inhibition, the estimated concentration of DHAP in field soil water should be well over the threshold of seed germination and growth inhibition. Therefore, DHAP in field soil likely inhibits the seed germination and seedling growth of* P. schrenkiana* and other co-occurring species although particular responses might depend on whether and how DHAP was maintained in soil solution. DHAP inhibition of *P. schrenkiana* recruitment might partly explain the relatively wide spaces between individuals in *P. schrenkiana* forests.

Chemical regulation of *P. schrenkiana* recruitment, as demonstrated in the study, suggested a dual role of DHAP as an allelochemical and an autotoxicant. In the native range of *P. schrenkiana* in Tianshan Mountains, the importance of DHAP as an allelochemical might be limited by DHAP resistance in Tianshan species. Thus, DHAP might be more important for self-regulation than for interspecific interference in Tianshan *P. schrenkiana* forests. Field examination of intraspecific chemical inhibition in Tianshan *P. schrenkiana* forests might yield further insights into the role of DHAP in the native range of *P. schrenkiana*. Furthermore, the mechanisms that DHAP induces growth stress and alters the biochemical and physiological processes needed to determined. Additionally, the role of fire to mitigate the effect of DHAP, and thus promote *P. schrenkiana* regeneration needs also to be studied.

## 4. Experimental

### 4.1. General

The active compound was analysed by LC-MSD, ^1^H- and ^13^C-NMR (Bruker Ac-400 spectrometer) and optical rotations were measured on a Rudolph Research Auto Pol IV polarimeter. An Agilent 1100 LC-MSD with an API 2000 triple-quadrupole mass spectrometer was used for the LC-MS analysis. Electrospray ionization (ESI) in the negative ion mode was used as the ionization source. Nitrogen was used as the nebulizer gas and was maintained at a flow of 10.0 L/min with a nebulizer pressure of 40 psi. The gas temperature was set at 350 °C and the capillary voltage was 3,000 V. The fragmentor voltage was set at 120 V and the gain was 2.0. For HPLC a ZORBAX sb-aq C18, 3.5 µm, 150 × 2.1 mm column was used. The mobile phase was composed of: (A) 0.5% acetic acid and (B) acetonitrile. The flow was 0.25 mL/min, and a gradient was used as follows: 10% B for 5 min, 40% B from 5 to 15 min. The injection volume was 10 µL.

The original water extract was diluted to concentration of 10% (V/V) in HPLC-grade methanol and loaded onto a Hitachi L-2000 HPLC instrument equipped with a C18 reversed-phase column (Varian Microsorb-MV 100-5 C18, 4.6 × 250 mm, 5 µm). The optimum efficiencies of separation were obtained using linear gradients of a mobile phase of acetonitrile-0.5% acetic acid starting from 10:90 and changing to 90:10 in 30 min. The hold time was 10 min. The flow rate was 1.0 mL/min at a 25 °C column temperature. The injection volume was 10 µL. Detection was performed using a diode array detector set at 278 nm.

### 4.2. Plant Materials

Needles and cones of *P. schrenkiana* were collected from five trees located at the Xinjiang Agricultural University (1890 m, 43°22' 58″ N/86°49' 33″ E) on September 15-22, 2008. All selected plants were 30–35 m tall, about 80–100 years old, healthy and without infection. After collection, the cones were dried in paper bags at room temperature for 7 d and then threshed by hand to get seeds. Ten soil samples were collected from the same filed site. Two soil samples were collected under each tree. Soil cores, 1 cm in diameter by 5 cm deep, were collected at 20-cm from the tree. Seeds of rice (*Oryza sativa* L.), wheat (*Triticum aestivum* L.), radish (*Raphanus sativus* L.), lettuce (*Latuca sativa* L.), cucumber (*Cucumis sativus* L.), mung bean (*Phaseolus radiatus* L.) were purchased from Hangzhou, China.

### 4.3. Extraction and Isolation of the Active Compound

*P. schrenkiana* needles (200 g dry weight) were ground and exhaustively extracted at room temperature for 48 h with distilled water at a concentration of 1 g per 20 mL. The mixture was then sieved through cheesecloth and squeezed to extract as much liquid as possible and to remove as much leaf matter as possible. The remaining liquid was vacuum-filtered through Whatman No 4 filter paper. The original water extract was sequentially extracted three times with the same volume of diethyl ether, ethyl acetate, and *n*-butanol. The original water extract, diethyl ether fraction, ethyl acetate fraction, *n*-butanol fraction, and the water residue after the organic solvent extraction were submitted to an autotoxic activity test. From this bioassay, the autotoxic activity was found to reside in the diethyl ether fraction. Bioassay-guided fractionation of the diethyl ether fraction (3.74 g) by silica gel column chromatography (180 g, silica gel 100–200 mesh, Merck), eluted with petroleum ether and petroleum ether with increasing amounts of ethyl acetate (10% per step, v/v) gave twenty-two fractions (F_1_–F_22_) on the basis of TLC analysis. The most interesting active fractions were combined (F_7_–F_8_), then analyzed by chromatography by using a silica gel column (Scharlau GE 0048) and eluted with petroleum ether: ethyl acetate (6:4) to afford two subfractions (F_01_–F_02_). Subfraction F_02_ was re-crystallized with petroleum ether: Ethyl acetate (6:4) mixture and gave a yellow crystal (986 mg).

The active compound present in diethyl ether fraction was identified as DHAP by LC-MS. The retention time and the mass spectra of authentic standard under the chosen chromatographic condition were also recorded. The active compound was identified by comparing its retention time and mass spectral data with the authentic standard. Its molecular formula was determined to be C_8_H_8_O_3_ and molecular weight is 152.15 (MH = 151.35) (Figure 1a and 1b). ^1^H-NMR (CD_3_OD, 400 MHz): *δ* 2.42 (m, 3H, COC*H*_3_), 3.89 (s, 2H, O*H*), 6.75 (m, 1H, Ar–*H*), 7.31 (m, 2H, Ar–*H*). ^13^C-NMR (CD_3_OD, 100 MHz) δ 25.9 (CO*C*H_3_), 114.5, 114.7, 122.6, 129.5, 144.5, 150.4 (Ar–*C*), 198.6 (*C*OCH_3_).

The crystal structure of chemicals was determined by using data collected at T = 223 (2) K with MoKα radiation on a Nonius KappaCCD diffractometer. Crystal experimental data: Crystal system, monoclinic, Space group P21/*c*, a = 7.9894(17), b = 5.4562(10), c = 16.240(3) Å, *α* = 90, *β* = 94.747(5), *γ* = 90(°), *V* (Å^3^) = 705.5(2), Z = 4, *D*_calc_ (g·cm^−3^) = 1.432, *μ* (mm^−1^) = 0.110, *F* (000) = 320, Crystal size (mm) = 0.49 × 0.37 × 0.18, Reflections collected 3385, Independent reflections 1310, GOF on *F*^2^ 1.075, *R* [*I* > 2*σ* (*I*)] 0.0462, *wR*0.1162.

### 4.4. Bioassay

Stock concentration solutions of 100 µM were prepared using pure DHAP in distilled water. Stock solution was diluted to concentrations of 10, 5, 2.5, 1, 0.5 and 0.1 mM as treatment solutions and 0 mM as control for bioassays (the concentrations of treatment solutions chosen according to the measurement of levels of DHAP in the soil). Seed germination and seedling growth experiments were done according to ISTA (1993) [46].

#### 4.4.1. Effects of DHAP on Seed Germination

One hundred surface-sterilized* P. schrenkiana* seeds were placed in each sterile Petri dish (15 cm diameter) lined with Whatman No 3 filter paper in replicates of three. Ten mL of the treatment solutions were added to each Petri dish. Petri dishes were placed in programmable illuminated incubator with an L/D cycle of 16 h/8 h and a temperature cycle of 20 °C/15 °C. Treatments were allotted in a complete randomized design, with three replicates for each treatment. Germination (radicle emergence) was measured 7 and 21 days after treatment.

To test the effects of DHAP on seed germination of rice, wheat, radish, lettuce, cucumber and mung bean, we conducted experiments similar to those described above, except that incubator conditions were an L/D cycle of 12 h/12 h and a temperature cycle of 25 °C/15 °C. Germination (radicle emergence) was measured 5 and 14 days after treatment for rice, 4 and 8 days after treatment for wheat, 4 and 10 days after treatment for radish, 4 and 7 days after treatment for lettuce, 4 and 8 days after treatment for cucumber, 5 and 7 days after treatment for mung bean.

#### 4.4.2. Effects of DHAP on Seedling Growth

Pre-germination of *P. schrenkiana* seeds were achieved in plastic boxes (20 × 15 × 10 cm) lined with Whatman #3 filter paper for 5–6 days until radicle emergence. One hundred successfully germinated seeds were placed in Petri dishes in three replicates and 10 mL treatment solutions were added to each Petri dish. Seedlings were incubated in programmable illuminated incubator (incubation conditions were the same as *P. schrenkiana* seed germination). Five seeds were randomly taken out from each Petri dish and the length of shoot and root were measured with a vernier caliper (GB/T 1214.2-1996, Measuring Instrument LTD, Shanghai). Fresh weight of seedlings was also recorded (Mettler Toledo Instrument Ltd). The measurements were taken on the third day after incubation, and continued once every three days for a total of 30 days.

Bioassays of DHAP on seedling growth of other six tested species were the same as above, expect that the programmable illuminated incubator conditions were an L/D cycle of 12 h/12 h and a temperature cycle of 25 °C/15 °C. Shoot, root length and fresh weight were measured every day after incubation, and continued for a total of 10 days. 

### 4.5. Quantify DHAP Content in Needles and Soil

*P. schrenkiana* needles (10 g dry weight) were ground and extracted with distilled water as described above. Field soil samples (10 g dry weight) were also extracted with distilled water as described above. The original water extract was diluted to concentration of 10% (V/V) in HPLC-grade methanol and loaded onto a Hitachi L-2000 HPLC instrument. The active compound in the needle sample was quantified by interpolating the peak areas on the HPLC chromatograms to a standard curve constructed by the peak height of authentic standard.

### 4.6. Statistical Analysis

We calculated germination rate and germination vigor for *P. schrenkiana* and each of the other six tested species. The number of seeds germinated within 21 (*P. schrenkiana)*, 14 (rice), eight (wheat and cucumber), 10 (radish), seven (lettuce and mung bean) days were used to calculate germination rate while the number of seeds germinated within seven (*P. schrenkiana)*, five (rice and mung bean), and four (wheat, cucumber, radish and lettuce) days were used to calculate germination vigor. The significant differences among treatment solutions and control on seed germination and seedling growth of *P. schrenkiana* and six test species were first examined by ANOVA (*p* < 0.05) and then analyzed using Fisher’s LSD test at *p* < 0.05 level.

## 5. Conclusions

Our results demonstrated that DHAP acted both as an autotoxicant and allelochemical because of its intraspecific and interspecific effects. Accumulation of DHAP in *P. schrenkiana* forest soils may have a negative impact on *P. schrenkiana* natural regeneration.
